# London Locomotion

**Published:** 1903-03-14

**Authors:** 


### London Locomotion.

The problem of London locomotion, which is about to be submitted to inquiry and report by a Royal Commission, embraces, in its widest sense, questions of the very highest importance to the health and welfare of the enormous population of the metropolis and of its suburbs, questions which cannot too soon receive the careful attention of the legislature. Sir John Wolfe-Barry has, we believe, made some rather surprising estimates as to the amount of the daily loss in money, as measured by the loss of time, which is occasioned by the street hindrances incidental to the ordinary amount of current traffic; and it would not be difficult to show that the same conditions must involve no inconsiderable waste or destruction of health. Few spectacles are more curious than that which is presented, every weekday, when the morning trains from the suburbs arrive at any of the great metropolitan termini. Before any such train comes to a standstill, its doors fly open simultaneously, and it disgorges some four hundred or five hundred passengers, of whom, say, between the hours of eight and nine, the majority are young men and women, and of whom nearly all break at once into a run. It may be that they are only eager to reach an omnibus in the station yard before it is filled up; but, in most cases, a daily ride would be too large a tax upon their slender earnings, and they run to, or far towards, their destinations in shops, warehouses, and offices. It would seem as if, as a rule, they had remained in their suburban homes, either for the sake of longer rest or in obedience to the claims of domestic duties, until the last moment which would permit them to arrive at business with the necessary punctuality; and, this being so, the smallest delay in the running of their trains changes what may be called the arrival expression of their faces from mere eagerness into acute anxiety. Of weather, of course, they can in such conditions take no heed; and they must often suffer, temporarily or permanently, from its inclemency. An hour later the arrivals are of a superior, or at least of a better paid character; and the stress upon them has either perceptibly diminished or altogether disappeared. Even to these, however, punctuality, if not essential, is at least important; and a wet morning entails a complete break down of their ordinary arrangements. For every cab which enters the station yard there will be half a dozen applicants, and the omnibuses will be filled up four or five deep as they stand waiting their turns to start. An organisation which is barely sufficient for the most favourable conditions practically disappears when the conditions become unfavourable; and the latter contingency, between our railway system and our climate, is too frequently fulfilled to be regarded as exceptional. On the return journey, especially at the omnibus and tramcar termini, matters are often worse than on arrival. Two or three would-be passengers are struggling for every vacant seat, while the conductors are helpless, and a casual policeman on the footpath regards the scene with tranquil amusement. The commercial Hooligan with a foul short pipe hustles or drags girls and women from their places, and bestows a final puff of smoke upon them as they are ejected.

It is abundantly manifest that the state of things thus described must detract largely from the health and comfort of those who are compelled, by the lack or the costliness of London habitations, to travel daily between some suburb and the scene of their employment, and it is equally manifest that the difficulties referred to are largely dependent upon a want of elasticity in the existing arrangements. At certain hours of the day, at certain seasons of the year, and in certain states of the weather, the demand for conveyances is at least twice that which is incidental to other hours, seasons, or states; and, as far as we can see, no attempt is ever made to recognise or to provide for the difference. The London County Council has a monopoly of the tramlines within its jurisdiction, and works those which run to the southern suburbs. The cars have practically driven other public conveyances off the roads. In fine weather they are sufficient for the requirements of the case; in wet weather it is hopeless to seek for a place at any intermediate part of their journey. They become filled up at or near their starting point. No attempt is made to increase the accommodation in order to meet any temporarily increased demand. The cars run perhaps every minute and a half or two minutes, and there is no reason why they should not follow each other in an almost continuous line. The very facilities which they have afforded, like all other facilities, have tended powerfully towards the permanent increase of demand for them, but no attempt has been made to meet this permanent increase. Perhaps when the lines are worked, as they soon will be, by electricity, more accommodation will be provided; but even then experience will soon show, as it has so often shown before, that nothing in London is too large to be overtaken by the requirements which it is designed to meet. An electric system ought at least to be more elastic than one dependent upon horses, but we have very little faith in the administrative capacity of the London County Council or its committees.

